# *BRAF* V600E mutation as a novel mechanism of acquired resistance to ALK inhibition in *ALK*-rearranged lung adenocarcinoma: A case report

**DOI:** 10.1097/MD.0000000000024917

**Published:** 2021-02-26

**Authors:** Aixia Sui, Huiling Song, Yitong Li, Litao Guo, Kai Wang, Mingming Yuan, Rongrong Chen

**Affiliations:** a Department of Oncology, Hebei General Hospital, Shijiazhuang, China; b Geneplus-Beijing, Beijing, China.

**Keywords:** ALK inhibition, *BRAF* V600E, case report, lung adenocarcinoma, resistance mechanism

## Abstract

**Rationale:**

Patients with lung adenocarcinoma harboring *EML4-ALK* rearrangements respond well to multiple ALK tyrosine kinase inhibitors (TKIs). However, the tumor will invariably progress due to acquired resistance. Comprehensive genomic profiling appears to be a promising strategy to reveal the underlying molecular mechanisms of ALK-TKIs resistance.

**Patient concerns:**

A patient with right lung adenocarcinoma harboring an *ALK* rearrangement received targeted therapy with multiple ALK-TKIs. He sought for follow-up treatment after his disease progressed again.

**Diagnosis:**

The patient had a tumor diagnosed with stage I (T1bN0M0) lung adenocarcinoma.

**Interventions:**

Due to the surgical contraindication, the patient did not undergo surgical resection. Instead, he received crizotinib as the first-line therapy with the progression-free survival of 20 months. Then he switched to alectinib treatment, however the disease rapidly progressed again.

**Outcomes:**

Next-generation sequencing was performed and revealed that 7 somatic mutations were identified. Among them, 2 mutations, *ALK* I1171T and *BRAF* V600E, may be responsible for the resistance of this patient to ALK-TKIs. *BRAF* V600E mutation may explain the patient's resistance to lorlatinib.

**Lessons:**

We present a case of *ALK*-rearranged lung adenocarcinoma with acquired resistance to ALK inhibition, in which the *BRAF* V600E mutation is a novel resistance mechanism. This provides evidence that *BRAF* V600E mutation is one mechanism of ALK-TKI resistance.

## 1 Introduction

Anaplastic lymphoma kinase (*ALK*) rearrangements, occurring in approximately 5% of lung adenocarcinomas, define a distinct molecular subtype of lung cancer.^[1–3]^ Patients harboring *EML4* (Echinoderm microtubule associated protein like 4)-*ALK* rearrangement, which is the most common type,^[3]^ respond well to multiple ALK-TKIs, including the first-in-class ALK-TKI (crizotinib), 3 second generation ALK inhibitors (ceritinib, alectinib, and brigatinib) and the third-generation ALK inhibitor (lorlatinib).^[4–8]^ These targeted drugs have significantly improved the prognosis of patients with *ALK* rearrangement.^[9]^ However, patients will invariably progress due to acquired resistance. Therefore, exploring the resistance mechanisms and developing strategies to overcome or prevent resistance have become an urgent priority. There are 2 major classes of ALK-TKI resistance mechanisms: ALK-dependent mechanisms including ALK secondary resistance mutations or amplification, where the tumor cell dependency on ALK signaling persists, and ALK-independent mechanisms including activation of bypass and downstream signaling, where the tumor cells effectively escape from dependency on ALK signaling.^[10]^

Using targeted next-generation sequencing (NGS) of 1021 cancer-related genes, we found the emergence of a *BRAF* V600E mutation in the peripheral blood of a patient with *ALK*-rearranged lung adenocarcinoma after treatment progression on ALK-TKIs, which may explain the resistance.

## 2 Case presentation

A 61-year-old Chinese male with T1bN0M0 (stage I) right lung adenocarcinoma harboring *ALK* and ROS proto-oncogene 1 (*ROS1*) double-rearrangements, due to the surgical contraindication, received crizotinib 250 mg twice daily as the first-line therapy with the progression-free survival of 20 months. Anti-bone metastasis therapy with zoledronic acid was added following progression on crizotinib treatment. Seven months later, the spoiled gradient recalled imaging of the skull revealed the presence of nodular hyperintensity in the sulci of the left occipital lobe, and the computed tomography pulmonary angiography indicated right pneumonia. At the same time, the patient has clinical symptoms such as the worsening of chest distress and asthma, showing disease progression. Two cycles of chemotherapy intrapleural infusion were added to control the worsening disease. The patient then switched to alectinib 600 mg twice daily treatment without genetic testing. Frustratingly, 3 months later, the patient had multiple bone metastases again. As a result, radiation and zoledronic acid were added. However, the disease has not been improved significantly, and progression has quickly occurred with emergence of multiple small nodules in both lungs, zoledronic acid and chemotherapy were rechallenged to control the progressive disease.

To identify potential molecular mechanisms of resistance and seek for other actionable mutations, NGS of 1021 cancer-related genes was performed using peripheral blood. Seven somatic mutations were identified, which were summarized in Table 1. The previous *ALK* rearrangement (*EML4-ALK* (E20:A20)) was detected with the mutant allele frequency of 8.8%, and the previous *ROS1* rearrangement detected by fluorescence in situ hybridization was disappeared. Furthermore, 2 mutations, *ALK* I1171T and *BRAF* V600E, may be responsible for the resistance of this patient to crizotinib and alectinib. Based on these results and the patient's preference, the patient chose lorlatinib for treatment. Unfortunately, the patient's condition did not improve, and died 3 weeks thereafter.

**Table 1:** Somatic mutations detected by next-generation sequencing.

Single-nucleotide variants				
Gene	Transcript	c.HGVS	p.HGVS	Allele Frequency
*BRAF*	NM_004333.4	c.1799T>A	p.V600E	8.3%
*EZH2*	NM_004456.4	c.186G>T	p.Q62H	8.0%
*SMARCB1*	NM_003073.3	c.94G>T	p.V32L	7.4%
*ASPM*	NM_018136.4	c.1505C>A	p.A502D	1.0%
*MLL3*	NM_170606.2	c.1072T>G	p.F358V	1.0%
*ALK*	NM_004304.4	c.3512T>C	p.I1171T	0.5%
*ERBB3*	NM_001982.3	c.1466C>A	p.P489Q	0.5%

Fusions			
Gene	Transcript	Functional Region	Allele Frequency
*EML4-ALK*	NM_004304.4; NM_019063.3	EX20:EX20	8.8%

HGVS = Human Genome Variation Society.

## 3 Discussion

Numerous clinical trials have documented the promising efficacy of ALK-TKIs in patients harboring *ALK* rearrangements.^[4–8]^ Although ALK-TKIs has significant responses, acquired resistance is still present at a later stage. After progression of ALK-TKIs, treatment choice can be guided by NGS testing for the identification of potential ALK-TKIs resistance mechanisms.

There are mainly 2 classes of ALK-TKIs resistance mechanisms: *ALK* dependent mechanisms and *ALK* independent mechanisms.^[10]^*ALK* dependent mechanisms include *ALK* secondary resistance mutations in the ALK kinase domain and *ALK* amplification.^[11,12]^ The *ALK* secondary resistance mutations induce kinase and signaling reactivation by altering the conformation and/or the ATP-binding affinity of the kinase, and thus preventing the binding of TKIs to the target kinase.^[11]^ About 20% to 30% of patients treated with crizotinib have the *ALK* secondary resistance mutations, compared to 50% to 70% receiving second generation ALK TKIs.^[10]^ Different ALK-TKIs have shown different sensitivity profiles to various resistance mutations. For example, *ALK* G1202R is a resistance mutation for the first and second generation ALK-TKIs, but lorlatinib, the third generation universal inhibitory ALK-TKI, has been shown to inhibit it effectively.^[10]^ In addition, patients harboring *ALK* I1171T mutations are resistant to crizotinib and alectinib, but sensitive to ceritinib and lorlatinib.^[10]^ In vitro studies have shown that lorlatinib can significantly inhibit the growth of cell lines carrying *ALK* I1171T mutations, and can cause the brain metastases with *ALK* I1171T to regress and prolong the survival of mice.^[13]^ Another study reported that, the lorlatinib-sensitive *ALK* resistance mutation I1171N was subsequently disappeared or no longer detectable in patients with non-small cell lung cancer (NSCLC) after treatment with lorlatinib.^[14]^*ALK* amplification was less frequent than *ALK* mutations, most commonly occurring following crizotinib therapy.^[12]^*ALK* independent mechanisms consist of the activation of bypass and downstream signaling activation, such as *EGFR* activation, *MET* amplification, *MEK* reactivation, as well as phenotypic changes such as epithelial-to-mesenchymal transition or small cell lung cancer transformation.^[10]^ In addition, an overexpression of P-glycoprotein (PGP), an efflux pump, was identified as a potential resistance mechanism.^[15]^ PGP was shown to be responsible for decreased concentration of ALK inhibitor (crizotinib or ceritinib) in brain tissues.^[15]^

In this study, 2 mutations, *ALK* I1171T and *BRAF* V600E, may be responsible for the resistance of this patient to ALK-TKIs. Among them, *ALK* I1171T mutation mediates resistance to crizotinib and alectinib, while may be sensitive to lorlatinib according to the results of preclinical studies.^[14,16]^ In our case, however, the patient did not respond to treatment with lorlatinib, which suggests that the lorlatinib resistance is more likely due to the *BRAF* V600E mutation. *BRAF* is an important gene in the RAS-MEK pathway, which was found to be the critical downstream effector of *ALK* signaling. The detection rate of *BRAF* mutation in *ALK* rearranged NSCLC is about 0.6%.^[17]^ In addition, one of 27 *ALK* positive patients who were resistant to ALK-TKIs had a *BRAF* mutation, according to the literature.^[16]^ The relationship between *BRAF* mutations and ALK-TKI resistance is not further elucidated in these studies. In our case, NGS results showed that the frequency of the *BRAF* V600E mutation was significantly higher than the *ALK* I1171T mutation, which may also explain the insensitivity of lorlatinib in this patient. In addition, the progression-free survival (PFS) of alectinib was 3 months, which is much lower than the median PFS of alectinib in crizotinib-resistant NSCLC (8 months) in previous clinical trial.^[18]^ As no genetic testing was performed before alectinib administration, we suspected that the *BRAF* V600E resistance mutation may have been present before the administration of alectinib and lead to a nondurable response to alectinib.

## 4 Conclusion

In conclusion, we analyzed the potential molecular mechanisms of drug resistance in a lung adenocarcinoma patient with *ALK* rearrangement following sequential *ALK*-TKIs treatment. This provides evidence that *BRAF* V600E mutation may be one of the mechanisms of *ALK*-TKIs resistance.

## Acknowledgments

We owe thanks to the patient and his family.

## Author contributions

All authors read and approved the final manuscript.

**Resources:** Aixia Sui.

**Supervision:** Aixia Sui.

**Validation:** Huiling Song, Litao Guo, Yitong Li.

**Visualization:** Rongrong Chen.

**Writing – original draft:** Kai Wang, Mingming Yuan.

**Writing – review & editing:** Aixia Sui, Rongrong Chen.
